# Stability of exploratory multivariate data modeling in longitudinal data

**DOI:** 10.1186/1471-2156-4-S1-S38

**Published:** 2003-12-31

**Authors:** Haydar Sengul, M Michael Barmada

**Affiliations:** 1 Department of Human Genetics, Graduate School of Public Health, University of Pittsburgh, Pittsburgh, Pennsylvania, USA

## Abstract

Exploratory data-driven multivariate analysis provides a means of investigating underlying structure in complex data. To explore the stability of multivariate data modeling, we have applied a common method of multivariate modeling (factor analysis) to the Genetic Analysis Workshop 13 (GAW13) Framingham Heart Study data. Given the longitudinal nature of the data, multivariate models were generated independently for a number of different time points (corresponding to cross-sectional clinic visits for the two cohorts), and compared. In addition, each multivariate model was used to generate factor scores, which were then used as a quantitative trait in variance component-based linkage analysis to investigate the stability of linkage signals over time. We found surprisingly good correlation between factor models (i.e., predicted factor structures), maximum LOD scores, and locations of maximum LOD scores (0.81< ρ <0.94 for factor scores; ρ >0.99 for peak locations; and 0.67< ρ <0.93 for peak LOD scores). Furthermore, the regions implicated by linkage analysis with these factor scores have also been observed in other studies, further validating our exploratory modeling.

## Background

When examining large amounts of data with many correlated variables, a common approach is to employ dimensionality-reducing techniques, such as clustering methods, principle component analysis, or common factor analysis. Each of these methods attempts to reduce the complexity in large systems by creating combinations of variables that reflect underlying, unobservable structures inherent in the data. Applying these methods to understand the structure of complex data falls into the realm of exploratory data analysis [1], and is commonly followed by a confirmatory phase, in which the reproducibility of the results is investigated.

Multivariable modeling of this type has been used to identify genetic latent factors in studies of twins and family data [2]. These genetic latent factors are thought to represent the effects of genes, which exhibit pleiotropic effects on the measured variables with which the factors are correlated. Several studies [2-4] have examined the effect that pleiotropy has on analysis methods and study design. These studies conclude that recognition of pleiotropic effects on multiple measured traits and application of appropriate multivariate analysis methods results in increased power to detect a genetic effect compared with univariate (simple) analysis of the individual measured traits.

In line with this approach, we have applied the method of factor analysis to investigate the underlying structure of data in the Framingham Heart Study (FHS) data provided as part of the Genetic Analysis Workshop 13 (GAW13) conference. The FHS represents one of the largest (and longest) ongoing projects in the history of NIH-funded research, with a goal of identifying risk factors that contribute to the development of cardiovascular disease. This objective was pursued by collecting a population-based sample and following them forward in time (an example of repeated-measures prospective study design). Since these data were collected longitudinally over many years, we chose to investigate the stability of factors by comparing factor models and resulting multipoint linkage signals generated from different time points.

## Results

Linear and logistic regressions were used to remove the effects of age and gender from all selected traits. Regressions were performed for each time-point independently. Residuals from each regression were used as inputs to factor analysis, thus generating four sets of factor structures (one for each time-point). The composition of the factors for each time-point are given in Table 1. Predicted factor scores were then generated using these models for all phenotyped individuals. Correlations between the factor scores are presented in Table 2. These factor scores, along with the pedigree information and marker genotypes from the FHS genome-wide scan were used in a variance component linkage analysis. A summary of the significant multipoint LOD scores, as well as their chromosomal locations, is given in Table 3.

## Discussion

Exploratory factor analysis allows the researcher to investigate the structure of complex data by looking for commonalities between variables. These commonalities become expressed as the function of an unobserved latent variable that manifests on the system in question through the measured variables with which it is correlated. This concept works well with classical genetics, which explains the latent variables as underlying genetic factors exhibiting pleiotropic effects on the system of measured traits.

In testing genetic factor models, a key issue is identifiability. Appropriately applied rotations can modify the loadings and the resulting factor scores, so that factor solutions are not unique. Thus, the factor loadings to be used in genetic modeling must correspond to what is known about the biology of the system under investigation. In our study, the first and third factors can be identified as components of lipid homestasis. The factor loadings for the first factor (see Table 1) have the highest degree of identifiability, and seem to describe a latent variable which is highly correlated with high-density lipoprotein (HDL) levels, and correspondingly inversely correlated with triglycerides, smoking, and body mass index (BMI). These loadings are in line with current understanding of the biology of high density lipoproteins. Thus, in some way, this factor can be thought of as having a significant effect on HDL levels (perhaps coding for a component of HDLs). Looking across timepoints, it appears that this definition remains fairly stable, differing only in later timepoints with the addition of blood pressure and fasting glucose as predictive factors, and the removal of smoking (perhaps as older individuals cease, or at the very least reduce, the smoking habit of their earlier years). It is interesting to note that, although apparently similar in factor loadings, the factors from the first and subsequent time points differ significantly, as exemplified by the correlation between factor scores (the correlation between time1 and other times' scores for this factor is low – on the order of 0.1–0.2, whereas the correlation between other times for this factor are fairly high – on the order of 0.8–0.9). This also explains the difference observed later in peak LOD scores and positions in time1. The exact source of this difference is unknown, but may be due to numerical instability in the factor solution for the first time point (the loading of 1.0 on HDL levels, and the concomitant communality of 1.0 for HDL as a variable, indicates the presence of a Heywood instability, or a problem with the factor solution).

Variance component-linkage analysis using the predicted factor scores highlights several regions of the genome. Most prominent in these analyses are the regions on chromosome 6 (at roughly 127 cM) and chromosome 7 (at 160 cM), which correspond with previous studies of HDL variability [5,6]. The stability of the location estimates for these peaks (and their LOD scores) are impressive, and lend considerable confirmatory evidence to support our multivariate modeling.

Factor 3 may also be related to some component of the cholesterol homeostasis mechanism, as it seems to be correlated with triglyceride levels, fasting glucose levels, HDL levels, BMI, and systolic blood pressure. As with Factor 1, correlations between the different time point factor models are high. This component appears to be distinct from that described by Factor 1 based on the location of linkage signals produced by the Factor 3 structures.

For the other two factors, identifiability is an issue. Factor 2 resembles to some degree Factor 1, in that it is composed of contributions from triglyceride levels, total cholesterol levels, and blood pressure measures. However, this factor does not provide any unique (or significant) linkage results on its own, in spite of good correlations between factor scores derived from each time point. These findings suggest a limit to the correlation between factor scores that will generate significant, reproducible findings. Based on the correlations in Table 2, correlations of 70% or more appear to be required to get reproducible linkage findings from factor models. In this way, the table of factor score correlations could possibly be useful in predicting the strength of further analysis.

Factor 4 also defies our attempts at identification, as no clear pattern is observable in the factor loadings. These factor models may actually demonstrate the effect of extraneous variables in a factor model, as extraneous variables should appear in factor structures independently of the other variables. Because this happens in at least two of the time points for this factor, it is possible that these structures simply represent the residual variation left in the system after the effects of other latent variables have been taken into account. Additionally, no clear, reproducible linkage signals appear.

## Conclusions

We have demonstrated, by the application of multivariate modeling to a longitudinal data set, good stability of the predicted factor models, and surprisingly good stability of the locations and LOD scores from linkage analyses using the predicted factor structure when that structure corresponds with previous knowledge about the system being studied. Thus, the use of factor models for exploratory data analysis appears to be a suitable tool for the dissection of complex traits, and can be used to identify genetic factors in longitudinal studies.

## Methods

### Framingham Heart Study

The FHS has been described in detail elsewhere [7]. For the purposes of the GAW13 workshop, phenotype data were provided for the first 40 years (from 1948 to 1988, or 21 exams) of follow up in Cohort 1 and the first 20 years (from 1971 to 1991, or 5 exams) of follow up in Cohort 2. The individuals available for study come from 330 of the largest pedigrees in the FHS sample. These pedigrees comprise 4692 subjects, of which 2885 were available for study. Additionally, we have genotype data for Marshfield Mapping Set 8A on 1702 of these individuals. For the purposes of this study, use of this data has been approved by the institutional review board of the University of Pittsburgh (IRB approval number 020481).

### Matching of timepoints for Cohort 1 and 2

Since the FHS data were collected as two separate cohorts, and since the clinic visits were not scheduled at the same time, we had to find a way to match timepoints (clinic visits) between the two cohorts. To get cross-sectional samples from these two cohorts we averaged the data from the years 1970 and 1972 in Cohort 1 (or used the value present if data was available for only one of these years) to match the data from the year 1971 in Cohort 2 for all the variables used in this study (this is referred to hereafter as time1 data). We used the same method for data from years 1978 and 1980 (time2), 1982 and 1984 (time3), and 1986 and 1988 (time4) in Cohort 1 to match the data from the years 1979, 1983, and 1987 in Cohort 2, respectively. Thus we created four artificial time points for our studies.

### Selection of traits and removal of environmental factors

We selected the following traits to use as main effects in our modeling: fasting triglycerides, fasting glucose, fasting HDL cholesterol, total cholesterol, BMI (calculated from height and weight data), systolic blood pressure, high blood pressure, number of grams of alcohol per day, and number of cigarettes smoked per day. For regression modeling, we first selected one phenotyped individual at random from each family in Cohort 2 (the amount of missing data in all available timepoints in Cohort 1 prohibited the use of these individuals for regression modeling). Age and gender adjustments were made using standard multiple linear regression techniques. Logistic regression was used to model high-blood pressure as a trait (thus, age- and gender-adjusted risk of high-blood pressure was the trait used in all further multivariate modeling). Logarithmic transformations were used when necessary to correct for non-normal trait distributions. Transformation was necessary for triglyceride levels, fasting glucose, and HDL cholesterol levels. Residuals from the regression analyses were used as inputs for factor analysis.

### Factor analysis

We used a minimum-volume ellipsoid covariance estimate to perform the factor analysis. Factor analyses were performed using the R statistical package [8]. To be able to perform a test of the hypothesis that the specified number of factors is adequate to explain the model, we used the maximum likelihood factor estimate. This provides a likelihood-based test which compares the probability that the number of factors specified is sufficient with the probability that more factors are required. In this way, we were able to compare the validity of one-factor, two-factor, three-factor, and four-factor models for the system of traits. Using the residuals from the regression equations formed for each observed variable to adjust for age and gender, we arrived at a common factor solution for each time point corresponding to Exams 1 to 4 for Cohort 2. Four factors were found by the analysis in each case. The factor models predicted by each of these factor analyses were then applied to all the data from Cohort 1 and Cohort 2 to obtain estimated factor scores for all phenotyped individuals. Factor scores were generated using a regression-based approximation method.

### Variance component analysis

Using factor scores as trait values, we ran multipoint variance-component analysis using the SOLAR package [9]. The standard polygenic model was used for analysis, which hypothesizes a single major gene, a background (uncorrelated) polygenic effect, and an uncorrelated environmental effect. Fine mapping was performed on chromosomes which displayed a LOD score signal in excess of 1.5.

### Correlations

Correlations between factor scores, peak multipoint LOD scores, and peak locations were all calculated using the R statistical package [8].

## Figures and Tables

**Table 1:** Factor loadings Numbers indicate the degree of correlation between factor and trait.

**Trait**	**time1**	**time2**	**time3**	**time4**
**Factor 1**				
Triglycerides	-0.368	-0.293	-0.291	0.773
Fasting glucose	0.384	0.174	0.155	0.311
HDL	1.000	0.975	0.966	-0.863
Total cholesterol	0.135	0.318	0.629	0.140
BMI	-0.100	-0.100	-0.377	0.466
Systolic blood pressure	0.162	0.174	0.209	0.205
Risk of high blood pressure	0.193	0.533	0.118	0.617
Alcohol consumption	0.116	0.217	0.336	-0.105
Cigarette consumption	-0.132	-0.203	-0.185	0.122
**Factor 2**				
Triglycerides	0.917	0.609	0.835	0.231
Fasting glucose	-	0.329	0.33	0.166
HDL	-	-0.155	-0.185	0.122
Total cholesterol	0.396	0.787	0.122	0.986
BMI	0.147	0.987	0.253	0.203
Systolic blood pressure	0.615	0.221	0.419	0.973
Risk of high blood pressure	0.612	-	0.669	0.13
Alcohol consumption	0.203	0.402	0.173	0.13
Cigarette consumption	0.137	0.332	0.142	-
**Factor 3**				
Triglycerides	0.154	0.216	0.226	0.116
Fasting glucose	-	0.202	0.363	0.145
HDL	-	0.128	-0.133	0.21
Total cholesterol	-	-	-	-
BMI	0.445	-	0.651	0.13
Systolic blood pressure	0.312	0.498	0.22	0.108
Risk of high blood pressure	-	-	-	-
Alcohol consumption	0.369	-	-	0.137
Cigarette consumption	-0.169	-	-0.328	-
**Factor 4**				
Triglycerides	-	0.134	-	-
Fasting glucose	-	-	-	-
HDL	-	-	0.12	0.264
Total cholesterol	-	-	-	-
BMI	-0.192	-	-	-
Systolic blood pressure	-	-	-	-
Risk of high blood pressure	-	-	-	-
Alcohol consumption	-	-	-	0.565
Cigarette consumption	0.337	-	-	-

**Table 2:** Correlation matrices for factor scores

	**Factor1**			**Factor2**		
		
	**time2**	**time3**	**time4**	**time2**	**time3**	**time4**
**time1**	0.12	-0.18	-0.16	0.13	-0.41	-0.64
**time2**		-0.81	-0.86		-0.18	-0.33
**time3**			0.94			0.58

	**Factor3**			**Factor4**		
		
	**time2**	**time3**	**time4**	**time2**	**time3**	**time4**

**time1**	-0.70	0.73	-0.44	-0.07	0.24	0.45
**time2**		-0.89	0.53		-0.17	-0.13
**time3**			-0.56			0.21

**Table 3:** Maximum multipoint LOD scores and locations for variance-component analysis

**Chromosome**	**Location (cM)**	**time1**	**time2**	**time3**	**time4**
**Factor1**					
1	40	0.00	0.85	0.92	1.60
2	122	2.53	0.00	0.00	0.00
6	125–130	2.63	2.60	1.96	1.73
7	85	0.01	1.11	1.76	2.18
	160	0.99	1.61	0.49	1.03
10	150–161	0.00	1.98	2.11	2.84
16	20	0.47	1.01	1.09	1.83
**Factor2**					
2	105	2.12	0.15	0.25	0.05
**Factor3**					
1	20–21	0.81	2.29	1.52	0.00
1	205	0.29	1.74	0.70	0.75
2	26–31	2.35	4.77	3.62	0.46
12	125–134	3.82	2.05	3.59	0.00
	140	3.87	1.97	2.14	0.00
13	59–60	2.69	3.08	3.44	0.67
15	45	0.41	3.50	1.95	0.76
16	119–120	0.04	2.26	1.89	0.09
17	10	0.80	2.84	4.25	0.74
19	4–5	0.22	2.58	2.52	0.00
	14–17	0.44	2.54	2.59	0.00
20	70	0.57	1.45	1.89	0.28
22	30	1.94	1.02	1.46	0.22
**Factor4**					
1	100	0.21	0.00	-	1.59
2	69	0.38	0.08	-	2.12
4	20	0.00	0.03	1.64	-
	165	0.00	1.57	-	-
6	135	1.56	0.00	-	-
	154	1.00	0.00	-	2.35
8	15	0.69	0.00	-	1.72
17	13	0.00	3.04	-	-
19	68	0.00	0.15	2.38	-
	85	0.21	0.82	-	1.77
20	30	0.00	0.07	1.92	-
